# Skeletal muscle morphology in sarcopenia defined using the EWGSOP criteria: findings from the Hertfordshire Sarcopenia Study (HSS)

**DOI:** 10.1186/s12877-015-0171-4

**Published:** 2015-12-18

**Authors:** H. P. Patel, M. C. White, L. Westbury, H. E. Syddall, P. J. Stephens, G. F. Clough, C. Cooper, A. A. Sayer

**Affiliations:** Academic Geriatric Medicine, University of Southampton, University Hospital Southampton FoundationTrust (UHSFT), Tremona Road, Southampton, SO16 6YD UK; Medical Research Council Lifecourse Epidemiology Unit, University of Southampton, UHSFT, Tremona Road, Southampton, SO16 6YD UK; National Institute for Health Research Southampton Biomedical Research Centre, University of Southampton and UHSFT, Tremona Road, Southampton, SO16 6YD UK; Institute for Developmental Sciences, University of Southampton, UHSFT, Tremona Road, Southampton, SO16 6YD UK; National Institute for Health Research Collaboration for Leadership in Applied Health Research and Care: Wessex, Academic Geriatric Medicine, University of Southampton, UHSFT, Tremona Road, Southampton, SO16 6YD UK; National Institute for Health Research Musculoskeletal Biomedical Research Unit, University of Oxford, Oxford, UK; Newcastle University Institute for Ageing and Institute of Health & Society, Newcastle University, Newcastle, UK

**Keywords:** Sarcopenia, Muscle morphology, Community dwelling older men

## Abstract

**Background:**

Sarcopenia is defined as the loss of muscle mass and function with age and is associated with decline in mobility, frailty, falls and mortality. There is considerable interest in understanding the underlying mechanisms. Our aim was to characterise muscle morphology changes associated with sarcopenia among community dwelling older men.

**Methods:**

One hundred and five men aged 68–76 years were recruited to the Hertfordshire Sarcopenia Study (HSS) for detailed characterisation of muscle including measures of muscle mass, strength and function. Muscle tissue was obtained from a biopsy of the *vastus lateralis* for 99 men and was processed for immunohistochemical studies to determine myofibre distribution and area, capillarisation and satellite cell (SC) density.

**Results:**

Six (6 %) men had sarcopenia as defined by the European Working Group on Sarcopenia in Older People (EWGSOP) criteria. These men had lower SC density (1.7 cells/mm^2^ vs 3.8 cells/mm^2^, *p* = 0.06) and lower SC/fibre ratio (0.02 vs 0.06, *p* = 0.06) than men without sarcopenia. Although men with sarcopenia tended to have smaller myofibres and lower capillary to fibre ratio, these relationships were not statistically significant.

**Conclusion:**

We have shown that there may be altered muscle morphology parameters in older men with sarcopenia. These results have the potential to help identify cell and molecular targets for therapeutic intervention. This work now requires extension to larger studies which also include women.

## Background

Sarcopenia is associated with adverse health outcomes and incurs a substantial health care cost [1–3]. Defined as the loss of skeletal muscle mass and function, sarcopenia is common in both men and women over the age of 65 across a range of healthcare settings [4]. For example, among community dwelling older people in the UK, prevalence rates for sarcopenia have been estimated at 4.6 % for men and 7.9 % for women [5]. Sarcopenia has been defined based on lean mass indices i.e., total lean mass, appendicular lean mass and muscle function i.e., grip strength or physical performance. Notable diagnostic algorithms include The European Working Group on Sarcopenia in Older People (EWGSOP) [6], The Foundation for the National Health Institutes of Health (FNIH) Sarcopenia Project [7], and the Asian Working Group for Sarcopenia (AWGS) [8], the latter definition to account for ethnic variations in muscle mass and muscle function.

There is considerable interest in understanding the mechanisms driving sarcopenia and there have been a number of small studies investigating morphological changes in skeletal muscle with increasing age. For example, it has been reported that between the sixth and ninth decades, myofibre size and number decrease in both men and women [9]. In addition there is type I and type II myofibre atrophy, as well as other changes in muscle morphology including the appearance of hybrid fibres, the presence of hypertrophied fibres and fibre type grouping [10–12]. Although not completely understood, several factors contribute to these morphological changes including, inflammation, denervation, oxidative stress, imbalance in protein synthesis and reduced satellite cell number and or function [13].

Studies of muscle morphology have rarely been population based and how these morphological changes relate to altered skeletal muscle mass, function and sarcopenia is unclear. The objective of this study was therefore to determine the relationship between muscle morphology and sarcopenia as defined using the European Working Group Sarcopenia in Older People (EWGSOP) criteria in a population based study of community dwelling older men.

## Methods

### Study participants

One hundred and five community dwelling older men aged 68–76 years who had participated in the UK Hertfordshire Cohort Study (HCS) [14] were involved in the Hertfordshire Sarcopenia Study (HSS) [15]. We characterised their muscle morphology and functional parameters and applied the European Working Group on Sarcopenia in Older People (EWGSOP) diagnostic algorithm to identify sarcopenia [2]. Inclusion and exclusion criteria and study methods have been previously described in detail [15]. The study received ethical approval from the Hertfordshire Research Ethics Committee, number 07/Q0204/68. Each participant gave written informed consent.

### Muscle biopsy

Percutaneous muscle biopsies of the *vastus lateralis* were conducted under local anaesthetic using a Weil-Blakesley conchotome [16]. One hundred and two participants were eligible for the procedure; three were ineligible as they were taking medication that might influence subsequent wound healing (*n* = 2) or predispose to haematoma formation (*n* = 1). Biopsies from a further three participants were not suitable for analysis. Thus, the final muscle biopsy analysis sample comprised 99 participants.

### Immunohistochemistry

**Fig. 1:**
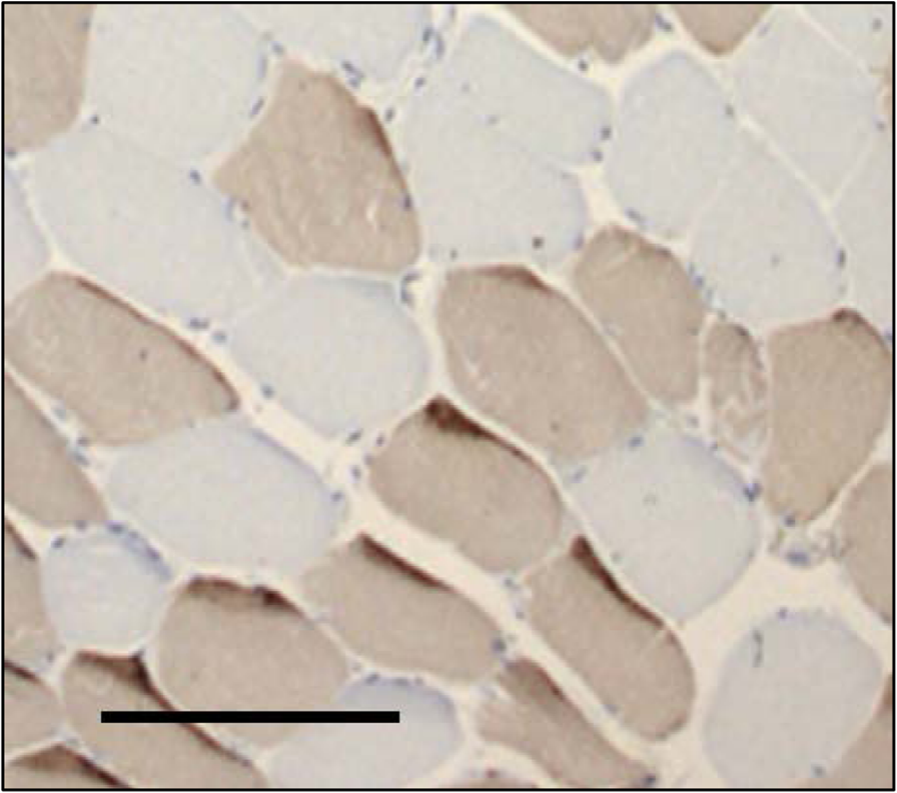
A serial cross section showing differential fibre staining. Darkly stained fibres represent type II, fast fibres (anti myosin-fast antibody, clone MY32, 1:6000 Sigma- Aldrich). Bar represents 200 μM

**Fig. 2:**
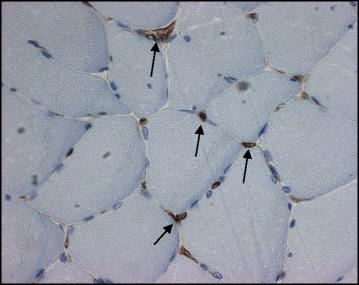
A serial cross section showing capillary staining. Capillaries are stained brown and are located at the peripheries of the myocyte. (Capillaries have been stained with Ulex Europeaus Agglutinin 1, 1:200, Vector laboratories, UK, visualised at magnification × 40 and are arrowed)

**Fig. 3:**
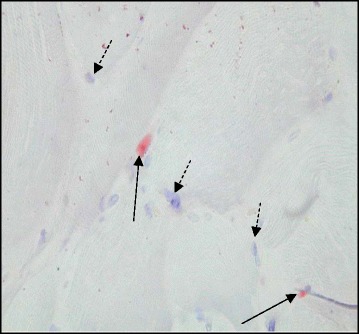
A serial cross section showing satellite cells (SC). SC have stained red at the peripheries of the myocyte and are marked with arrows. Myonuclei have been counterstained with Mayer’s hematoxylin and are marked with broken arrows (SC have been stained with PAX-7, Developmental Studies Hybridoma Bank, Iowa, 1:10, magnification × 40)

Muscle tissue was fixed overnight at −20 °C before being embedded in glycol methacrylate resin [17]. Serial cross-sections at 7 μm were cut and stained for type II fast-twitch myofibres using the monoclonal anti-myosin fast antibody at a dilution of 1:6000 (clone MY-32; Sigma-Aldrich, Dorset, UK) (Fig. 1). Capillaries were stained by incubating separate slides with biotinylated Lectin *Ulex Europeaus Agglutinin* 1 (UEA-1, Vector Laboratories, Peterborough, UK) at a dilution of 1:200 for two hours (Fig. 2). Stained sections were examined under a photomicroscope (Zeiss Axioskop II, Carl Ziess Ltd, Welwyn Garden City, UK) coupled to KS 400 image analysis software (Image Associates, Bicester, UK). Sections were viewed at a × 5 magnification and digitized to obtain tissue area, myofibre number (type I, slow fibre vs type II, fast fibre) and myofibre cross-sectional areas (μm^2^). Slow and fast fibre proportions were expressed as a percentage of total fibres. For capillaries, a digital image at a magnification of ×40 was taken of the section. The total number of muscle fibres and capillaries was quantified from the whole tissue area manually from the digital image. For each section, capillary density (capillaries per mm^2^) and capillary: fibre ratio was calculated. Satellite cells (SCs) were identified in separate tissue sections using a similar immunohistochemistry protocol with the primary antibody PAX-7 (Paired-box transcription-factor 7, Developmental Studies Hybridoma bank, University of Iowa) [18] (Fig. 3). SCs were identified by light microscopy and quantified (SC density [cells/mm^2^] and SC to fibre ratio) using image analysis techniques as described above. Muscle morphology parameters were analysed in all samples by a blinded observer.

### Defining sarcopenia using the EWGSOP criteria

For a diagnosis of sarcopenia, the EWGSOP recommend the presence of both low muscle mass and low muscle function as measured by strength and or muscle performance [2]. We use this definition based on our previous published work detailing the prevalence of sarcopenia in this cohort [5]. In our study, lean muscle mass was determined by dual-energy x-ray absorptiometry scanning (DXA; Hologic Discovery, auto whole body software version 12.5). Isometric maximum grip strength was identified from three measurements in each hand using a standardised protocol and Jamar dynamometer [18]. A validated battery of physical performance tests was administered which included time to complete 5 chair rises and measurement of customary gait speed over 3 m [19]. We used values in the lowest third of the distributions of DXA derived lean mass and gait speed, and grip strength values of <20 kg for women and <30 kg for men, within the EWGSOP diagnostic algorithm for sarcopenia [5].

### Statistical analysis

Normally distributed variables were summarised using means and standard deviations (SD). Skewed variables were log_e_ transformed to normal distributions as necessary; for these variables, means and SDs on the log_e_ scale were back transformed to geometric means and SDs on the original scale of measurement. Student’s t test was used to compare age, body size, physical performance, cardiovascular fitness and fibre morphology variables between those without, and those with sarcopenia. All analyses were carried out using Stata release 13 (StataCorp, Texas, USA). A p value of <0.05 was considered statistically significant.

## Results

### Participant characteristics

Six men had sarcopenia (6.2 % of the 97 men with complete data for items comprising the EWGSOP definition). Descriptive statistics for participant characteristics and fibre morphology in terms of satellite cell density (cell/mm^2^), satellite to fibre ratio type I and type II fibre distribution (%), size/area (μm^2^), capillary density (capillaries/mm^2^) and capillary to fibre ratio are presented in Table 1. The total number of fibres counted to determine the capillary and satellite cell indices is also presented in Table 1.

**Table 1:** Participant characteristics

	N	Mean (SD)	Min	Max
Age (yrs)	99	72.4 (2.4)	68.3	76.8
Height (cm)	99	174.1 (6.5)	157.6	193.7
Weight (kg)	99	82.7 (12.6)	58.1	118.8
DXA lean mass (kg)	99	56.4 (6.5)	42.7	75.9
Grip strength (kg)	99	38.7 (8.1)	18.0	66.0
Walking speed (m/s)^a^	97	1.1 (0.2)	0.5	1.6
Chair rise time (s)^b^	96	17.1 (4.0)	8.3	27.2
Diagnosis of Sarcopenia	97	6 (6.2 %)		
Fibre morphology				
Satellite cell density (cell/ mm^2^)^c^	69	3.6 (2.5)	0.0	26.4
Satellite cell to fibre ratio	69	0.05 (0.04)	0.00	0.18
Fibre counts	69	189.5 (1.96)	23.0	723.0
Slow-fibre proportion (%)	99	43.9 (13.1)	18.9	88.3
Fast-fibre proportion (%)	99	56.1 (13.1)	11.7	81.1
Slow fibre area (μm^2^)^d^	95	4775 (1202)	2059	9868
Fast fibre area (μm^2^)^d^	95	3953 (1144)	1843	7424
Capillary density (capillary/mm^2^)^e^	96	146.5 (43.2)	57.1	254.1
Capillary to fibre ratio^e^	96	1.3 (0.3)	0.6	2.2
Fibre counts	96	248.5 (110.5)	44.0	577.00

^a^Data not available on two participants

^b^Data not available on three participants

^c^Geometric mean (SD), 30 slides were unsuitable due to suboptimal SC staining

^d^4 slides were unsuitable for fibre area determination

^e^3 slides were unsuitable due to suboptimal capillary staining

### Relationships between fibre morphology and sarcopenia

We have previously reported on the relationships between sarcopenia and age, anthropometry and muscle function [5]. Men with sarcopenia tended to have lower SC density (1.7 cells/mm^2^ vs 3.8 cells/mm^2^, *p* = 0.06) and lower SC/fibre ratio (0.02 vs 0.06, *p* = 0.06) than men without sarcopenia (Table 2). Men with sarcopenia also tended to have, on average, smaller slow and fast fibre areas and lower capillary to fibre ratios. However, these morphological relationships were not statistically significant (Table 2).

**Table 2:** Muscle morphology according to sarcopenia status among community dwelling older men

	No sarcopenia		Sarcopenia		*P* value**
	N	Mean (SD)	N	Mean (SD)	
Fibre morphology					
Satellite cell density (cell/ mm^2^) ^a^	63	3.8 (2.4)	6	1.7 (2.2)	0.061
Satellite cells to fibre ratio	63	0.06 (0.04)	6	0.02 (0.02)	0.063
Fibre counts	63	187.9 (1.96)	6	207.3 (2.07)	-
Slow fibre proportion (%)	91	44.3 (12.4)	6	40.8 (24.0)	0.530
Fast fibre proportion (%)	91	55.7 (12.4)	6	59.2 (24.0)	0.530
Slow fibre area (μm^2^)	88	4820 (1198)	5	4113 (1122)	0.202
Fast fibre area (μm^2^)	88	3960 (1115)	5	3397 (1363)	0.280
Capillary density (capillary/mm^2^)	88	146.9 (42.8)	6	150.2 (55.3)	0.856
Capillary: fibre ratio	88	1.3 (0.3)	6	1.2 (0.4)	0.493
Fibre counts	88	241.9 (105.0)	6	350.8 (164.0)	-

^a^Geometric mean (SD)

** *p* value for t-test between non sarcopenic and sarcopenic individuals as defined by the EWGSOP criteria

Sarcopenia status could not to be determined for two participants because of missing walking speed data hence maximum sample size of 97

## Discussion

We have investigated the association between skeletal muscle morphology and sarcopenia defined by the EWGSOP criteria in a population based study of community dwelling older men. In this study, there was a suggestion that the six HSS men with sarcopenia had lower average SC density and SC/fibre ratio in comparison with those without sarcopenia but the relationship was not significant at the 5 % level.

Satellite cells (SC) are undifferentiated stem cells responsible for myofibre maintenance and are central to the growth and repair of muscle and have the ability to enter the cell cycle, proliferate and self-renew [20, 21]. SC number fluctuate in younger and older adults in response to both intrinsic and extrinsic regulatory cues [22]. For example, physical activity [23] as well as with drug treatment [24]. The niche surrounding the SC is also a critical regulator of function and is governed by growth factors, signalling molecules as well as innervation [22, 25].

In support of our results, satellite cell content of fibres has been reported to decrease in the muscles of older humans. For example, Verdijk et al. showed that in older humans, SC content, specifically in type II myofibres, were lower when compared to younger controls [22]. In a study by Kadi et al. SC per fibre ratio was significantly lower in healthy older men and women compared to their younger counterparts [26]. In the later study, no inference was made on whether there was a fibre specific reduction as was reported by Verdijk et al. Therefore it appears that a reduction in satellite cells may mediate the observed muscle atrophy, specifically of type II fibres, with age. However, a detailed morphological study conducted by Purves-Smith et al. suggested that in very old individuals who may have severe muscle atrophy, both myofibre types show atrophic changes [12].

The exact reasons for a decrease in SC content or a decline in SC function with age are unknown. Contributing factors include alterations in the surrounding environment including denervation and oxidative damage [27] as well as decrease in activity of crucial myogenic regulatory factors coupled with an increase in negative regulators of muscle growth [28, 29]. For example, myostatin appears to suppress certain myogenic regulatory factors crucial for proliferation and differentiation and therefore has been postulated to impair function as well as self-renewal of satellite cells [30]. Taken together, it appears that loss of SC or reduced SC function diminishes the ability of ageing muscle to both hypertrophy in response to stimulus or repair and self-renew in response to injury thereby contributing to sarcopenia [27]. However, a recent study in older mice who were SC deplete suggested that neither force generation nor single fibre cross sectional area was affected by a reduction in SC number [31]. Clearly, further studies are needed in both male and female ageing cohorts to investigate the role of SC in sarcopenia.

We observed a non-significant trend (*p* > 0.05) for men with sarcopenia to have, on average, smaller slow and fast fibre areas and lower capillary to fibre ratios. Whereas myofibre morphology measurements in relation to exercise, immobilisation and ageing have been described in a number of studies [9], this is one of the first studies to describe muscle morphology in relation to sarcopenia in community dwelling older men. Muscle mass and cross sectional area (CSA) are functions of myofibre size and number [32] and we speculate that smaller fibres seen in our study are associated with the decrease in total lean mass in men who were sarcopenic. The results of a longitudinal study by Frontera et al. [33] which revealed age related reductions in muscle cross sectional area as well as capillary to fibre ratio in men is in partial support of our cross sectional findings.

Our study had several limitations. First, the sample size was modest which will have limited statistical power. Second, the immunohistochemical methodology used to quantify the morphology parameters was open to observer error although consistent and rigorous methods were applied throughout the study in order to limit this possibility. Also, we did not evaluate SC content per specific fibre type or other indices of muscle capillarity [34]. Finally, the parameters measured may not accurately reflect the morphological changes occurring in muscle. Longitudinal studies would be helpful to more fully characterise the morphological changes that occur over time in the muscle of people with sarcopenia.

However, our study has a number of strengths. First, we have shown that it is feasible to obtain tissue from community dwelling older men in the context of an epidemiological birth cohort. The advantage of this is that morphological data can be combined with the extensive phenotypic data that has already been collected. Second, the immunohistochemical methodologies employed were based on tested protocols and can be applied to future large scale studies. For example, whereas SC have typically been studied through electron microscopy and immunofluorescence [35], few studies have employed simple immunohistochemistry techniques to quantify satellite cells in healthy individuals [26]. Our study shows that quantification of several morphological variables is possible using these methods. However, measurements of specific fibre type/SC content as well as other indices of capillarisation will need to be considered when applied to future studies.

## Conclusions

Our results suggest that men with sarcopenia may have decreased satellite cell content and perhaps also smaller fibres and lower capillary to fibre ratios in comparison with men without sarcopenia. We can only speculate, given the sample size and cross sectional nature of the study, that the morphological results seen not only have an effect on muscle mass but also on muscle quality [31]. These morphological changes impact on muscle performance in men with sarcopenia, who by definition have grip strengths of less than 30 kg, walking speed less than 0.8 m per second and low muscle mass [5].

The importance of this work is that the identification of morphological changes in older people with sarcopenia has the potential to identify cellular and molecular targets for therapeutic intervention. Methodological findings from this study now need to be applied to large scale studies that also include women.
